# C-Gait for Detecting Freezing of Gait in the Early to Middle Stages of Parkinson’s Disease: A Model Prediction Study

**DOI:** 10.3389/fnhum.2021.621977

**Published:** 2021-03-22

**Authors:** Zi-Yan Chen, Hong-Jiao Yan, Lin Qi, Qiao-Xia Zhen, Cui Liu, Ping Wang, Yong-Hong Liu, Rui-Dan Wang, Yan-Jun Liu, Jin-Ping Fang, Yuan Su, Xiao-Yan Yan, Ai-Xian Liu, Jianing Xi, Boyan Fang

**Affiliations:** ^1^Beijing Rehabilitation Hospital, Capital Medical University, Beijing, China; ^2^Peking University Clinical Research Institute, Beijing, China

**Keywords:** Parkinson’s disease, C-Gait assessment, walking adaptability, freezing of gait, rehabilitation

## Abstract

**Objective:**

Efficient methods for assessing walking adaptability in individuals with Parkinson’s disease (PD) are urgently needed. Therefore, this study aimed to assess C-Gait for detecting freezing of gait (FOG) in patients with early- to middle-stage PD.

**Method:**

People with PD (PWP) diagnosis (Hoehn and Yahr stages 1–3) were recruited from April 2019 to November 2019 in Beijing Rehabilitation Hospital. The participants performed six items of walking adaptability on an instrumented treadmill augmented with visual targets and obstacles (C-Mill). The patient’s walking adaptability was evaluated by C-Gait assessment and traditional walking tests, and FOG-related indexes were collected as outcome measures. Two discriminant models were established by stepwise discriminant analysis; area under the receiver operating characteristic (ROC) curve (AUC) was used to validate the models.

**Result:**

In total, 53 patients were included in this study. Most C-Gait assessment items had no or low correlations with traditional walking tests. The obstacle avoidance (*r* = −0.639, *P* = 0.003) and speed of adaptation (*r* = −0.486, *P* = 0.035) items could lead to FOG with high sensitivity. In addition, the C-Gait assessment model (AUC = 0.755) had slightly better discrimination of freezers from non-freezers compared with traditional walking test models (AUC = 0.672); specifically, obstacle avoidance and speed of adaptation have uniquely discriminant potential.

**Conclusion:**

C-gait assessment could provide additional value to the traditional walking tests for PD. Gait adaptability assessment, as measured by C-Gait, may be able to help identify freezers in a PD population.

## Graphical Abstract

Patients with early- to middle-stage PD (Hoehn and Yahr stages 1–3) were enrolled for C-Gait assessment and traditional walking ability assessments. The correlation of C-Gait assessment and traditional walking tests were studied. Two models were established based on C-Gait assessment and traditional walking tests to explore the value of C-Gait assessment in predicting freezing of gait.

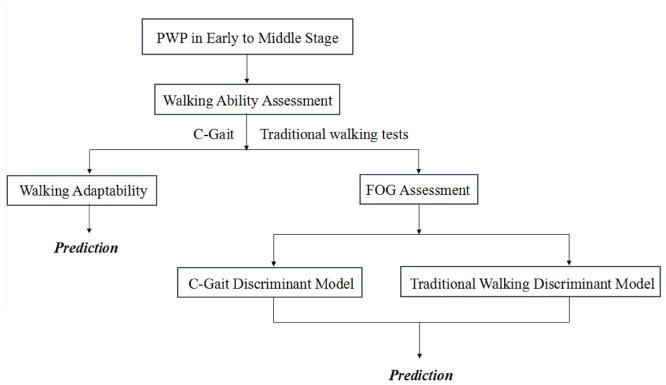

## Introduction

Parkinson’s disease (PD), a progressive neurodegenerative disorder commonly affecting middle-aged and elderly people, is found in 1–2% and 3–5% of individuals above 65 and 85 years, respectively, indicating that aging increases PD incidence (Alves et al., 2008). Freezing of gait (FOG) is a transient inability to produce effective stepping due to PD or identifiable causes; FOG can be provoked by many conditions, such as space obstacles and dual missions (Kader et al., 2017). Studies have shown that common motor symptoms of PD and FOG often impair the walking function (Kader et al., 2017; Opara et al., 2017). From a previous study, we know that the Parkinson’s patients’ walking ability, especially under a challenging environment, will become worse and worse when the disease gets into the late phase. This will consequently become a huge risk factor that will cause falls or even more severe situations (Caetano et al., 2017). Therefore, quantifying walking adaptability under complex conditions in early- to middle-stage PD could help guide the clinical management of patient activities of daily living, particularly in freezers.

Currently, most traditional walking tests in PD assess only a single aspect of walking ability, e.g., gait or posture and balance control (Wrisley et al., 2004; Bloem et al., 2016). For assessing FOG, the commonly used Freezing of Gait Questionnaire (FOG-Q) and New Freezing of Gait Questionnaire (NFOG-Q) have no correlations with FOG severity (Shine et al., 2012), not objectively reflecting walking adaptability. Balasubramanian et al. (2014) have provided a uniform definition for walking adaptability, whose clinical assessments must be comprehensive to encompass the multiple challenges of daily walking. Recently, virtual reality and the Interactive Walkway have been applied for assessing this ability in PD (Geerse et al., 2018), with insurmountable limitations (difficult implementation and patient safety). Consequently, efficient clinical methods for assessing walking adaptability in PD are urgently needed.

C-Gait assessment, designed for walking adaptability measurement with augmented-reality technology, can recreate the various complex tasks of walking and simulate real-life situations provoking FOG. It has good surface validity in individuals with different walking abilities, and most task items capture unique aspects of walking adaptability (Timmermans et al., 2019). However, the latter was a small-sample study not including PD cases, indicating that various domains in walking adaptability need to be further clarified. Therefore, this study aimed to evaluate C-Gait for detecting FOG in patients with early- to middle-stage PD.

## Materials and Methods

### Participants

This study is part of a prospective study (Active and passive biofeedback and neuromodulation collaborative therapy system evaluation and clinical validation, ChiCTR1900020771, registered on January 19, 2019). Patients with early- to middle-stage PD in the neurorehabilitation center of Beijing Rehabilitation Hospital from April 2019 to November 2019 were enrolled. Inclusion criteria were meeting the Movement Disorder Society (MDS) diagnostic criteria for primary PD (Postuma et al., 2015) in early to middle stages [Hoehn and Yahr stages 1–3 (Hoehn and Yahr, 1967)] during the “on” state; <75 years old; comorbidities not requiring special in-hospital treatment; no deep brain stimulation (DBS) therapy and no implantable medical device; Mini-Mental State Examination (MMSE) score >24 (education level ≥ secondary school) or >20 (≤elementary school) (Folstein et al., 1975); ability to stand unsupported for >20 s and walk independently (Geerse et al., 2018). Exclusion criteria were other diseases impairing walking ability, severe cognitive impairment affecting comprehension, antipsychotic drug use, considerable visual or auditory deficit, or combined serious complications contraindicated to rehabilitation. According to NFOG-Q (the patient’s presence or absence of FOG), all patients are divided into freezers (with FOG) and non-freezers. This study was approved by the Ethics Committee of Beijing Rehabilitation Hospital of Capital Medical University (No. 2018bkky022) and conformed to the Declaration of Helsinki. Written informed consent was obtained from all patients.

### C-Gait Assessment

“C-Gait” is an automatized and standardized assessment program supported by C-Mill (Motek, Netherlands) (Supplementary Figure 1A), comprising seven distinct items (Timmermans et al., 2019). Here, six items were applied, including Visually guided stepping, Tandem walking, Obstacle avoidance, Slalom walking, Reaction to unexpected perturbation, and Speed of adaptation (Supplementary Figure 1B and Supplementary Table 1). Because Cognitive Dual Tasking was not available in Chinese and hard to execute, it was excluded.

### Experiment Procedure

Before a formal study, patients need to be instructed to ensure that they can independently participate in the evaluation process. Guided by a well-trained therapist, the patient wearing the suspension protection device stepped onto the belt and started to warm up. The speed was determined by slowly increasing the belt speed in presumably 0.1 km/h until the subject reported feeling consistent with walking on the ground. After adaptation, six C-Gait assessment sessions were performed per patient. Overall assessment was performed at low and high difficulty levels. Baseline C-Gait assessment comprises the six items evaluated at level 2 (low difficulty level) and level 4 (high difficulty level), respectively. All patients were tested during the “on” state of the medication cycle, within 1–2 h after taking medicine. C-Gait assessment was administered for the second time on consecutive days on approximately the same time of the day. We adopted the second data as the assessment results so as to avoid errors caused by unfamiliar equipment. The whole assessment lasted for about 20 min.

In addition, the NFOG-Q (Nieuwboer et al., 2009) was used to evaluate FOG, and five traditional walking tests for gait, balance, and motor symptom assessments in PD were administered: 6-Minute Walk Test (6-MWT) (Bloem et al., 2016), Five-Times-Sit-to-Stand Test (FTSST) (Duncan et al., 2011), 10-Meter Walking Tests at self-selected (10-MWT-Selected) and maximum (10-MWT-Max) speeds (Bloem et al., 2016), and Timed Up-and-Go test (TUG) (Bloem et al., 2016); 10-MWT-Selected, 10-MWT-Max, FTSST, and TUG were repeated three times at 1-min intervals.

### Data Preprocessing

Outcome measures of the C-Gait baseline assessment comprised its belt speed and the composite score, performance per walking adaptability task, and overall performance averaged over the seven walking adaptability tasks, as detailed here (Timmermans et al., 2019). Task performance of slalom walking, tandem walking, speed adaptations, goal-directed stepping, obstacle avoidance, and walking with suddenly shifting obstacles and targets was defined as the percentage of correctly performed steps relative to the projected visual objects [based on center of pressure (COP) at mid-stance]. For instance, obstacle avoidance was presented as Missed (successfully avoiding the obstacle, with the distance between toe/heel and obstacle’s edge >3 cm), Near [1] [toe/heel close to but not touching the obstacle’s edge (distance <3 cm)], Near [2] (toe/heel stepping into the obstacle’s edge), and Hit (foot/shoe overlapping with the obstacle). Missed and Near [1] were recorded as “0” (successful obstacle avoidance) and Near [2] and Hit as “1” (stepping obstacles). The performance was assessed as (responses recorded as “0”/total responses) × 100%.

The six items were evaluated at low difficulty levels (defined as level 2) and high difficulty levels (defined as level 4). Only by completing both parts of the tests, we can get the result of each task. The composite score of the baseline assessment was an average score based on average performance over the six walking adaptability tasks at the higher level of difficulty. The composite score of the baseline assessment ranged from 0 (poor performance) to 8 (excellent performance).

• C-Gait score for each task: Level × 2 × Performance (%)/100
• C-Gait composite score: Mean C-Gait scores on high level.

### Establishment of the Model

Five traditional clinical assessments including 10-MWT-Selected, 10-MWT-Max, FTSST, 6MWT, and TUG were used as the original data set for establishing discriminant model 1 (DM1), and six tasks of C-Gait were used as the original data set for establishing discriminant model 2 (DM2). C-Gait assessment and traditional walking tests were transformed by the Tukey’s formula for normalization, and stepwise discriminant analysis was used to develop discriminant models, respectively, by the Wilks’ lambda method (entry = 3.84 and removal = 2.71) (Geerse et al., 2018). A variable can be included in the model only if its value of F to be added is greater than 3.84, and only a variable less than 2.71 can be moved out of the model.

### Statistical Analysis

SPSS 26.0 (IBM Corp., Armonk, NY, United States) was used for data analysis. Quantitative data were expressed as mean ± standard deviation (SD), and qualitative data were shown as count or percentage (%). The Shapiro–Wilk test was performed to assess normality, and normally and non-normally distributed variables were assessed by independent-samples *t*-test and Mann–Whitney *U* test, respectively. Associations of C-Gait assessment items with traditional walking tests were analyzed by the Spearman’s method.

DM1 and DM2 total scores as predictors of FOG were used to generate receiver operating characteristic (ROC) curve as an assessment of the accuracy of FOG predictions. The area under the ROC curve (AUC or c-statistic) ranges from 0 to 1.0, with an AUC of 0.5 indicating random chance and an AUC of 1.0 representing perfect accuracy. A general interpretation of an AUC value higher than 0.7 is considered good. *P* < 0.05 was considered statistically significant.

## Results

### Patient Characteristics

In total, 53 patients were enrolled in this study. Freezers (19 patients, 35.8%) and non-freezers (34 patients, 64.2%) showed no significant differences in sex, age, Hoehn–Yahr stage, and cognitive levels (*P* > 0.05; Table 1). However, freezers had significantly elevated Unified Parkinson’s Disease Rating Scale Part III (UPDRS-III) scores compared with non-freezers (*P* > 0.05; Table 1).

**TABLE 1:** Characteristics of PD patients and difference analysis of the freezer and non-freezer groups.

Variable	Total subjects (*N* = 53)	Freezers (*N* = 19)	Non-freezers (*N* = 34)	*P*
Sex^a^				0.422
Male	29 (54.7%)	9 (47.4%)	20 (58.8%)	–
Female	24 (45.3%)	10 (52.6%)	14 (41.2%)	–
Age^b^ (years)	60.34 ± 7.42	62 ± 5.77	59.41 ± 8.13	0.273
Disease duration^b^ (years)	6.00 (5.00)	6.00 (6.00)	5.00 (5.00)	0.600
Hoehn–Yahr stage^b^	2.50 (1.00)	3.00 (1.00)	2.25 (1.00)	0.072
UPDRS-III^c^ (score)	29.09 ± 10.40	34.53 ± 9.76	26.06 ± 9.59	0.003*
MMSE^b^ (score)	27.92 ± 1.67	27.68 ± 1.67	28.06 ± 1.69	0.404
NFOG-Q (score)	–	7.95 ± 5.83	0	–

*Quantitative data display as mean ± standard deviation. Qualitative data (sex) display as *N* (%). Disease duration and Hoehn–Yahr stage are reported as median (interquartile range). PD, Parkinson’s disease; UPDRS-III, Unified Parkinson’s Disease Rating Scale Part III; MMSE, Mini-Mental State Examination; NFOG-Q, The New Freezing of Gait Questionnaire.*

*^*a*^Difference in sex between freezers and non-freezers was assessed by the chi-square test.*

*^*b*^Differences in age, disease duration, Hoehn–Yahr stage, and MMSE between freezers and non-freezers were assessed by the Mann–Whitney *U* test.*

^*c*^Difference in UPDRS-III between freezers and non-freezers was evaluated by independent-samples *t*-test.

*meaning P < 0.05.

### Correlation of C-Gait Assessment With Traditional Walking Tests in Parkinson’s Disease Patients

**FIGURE 1:**
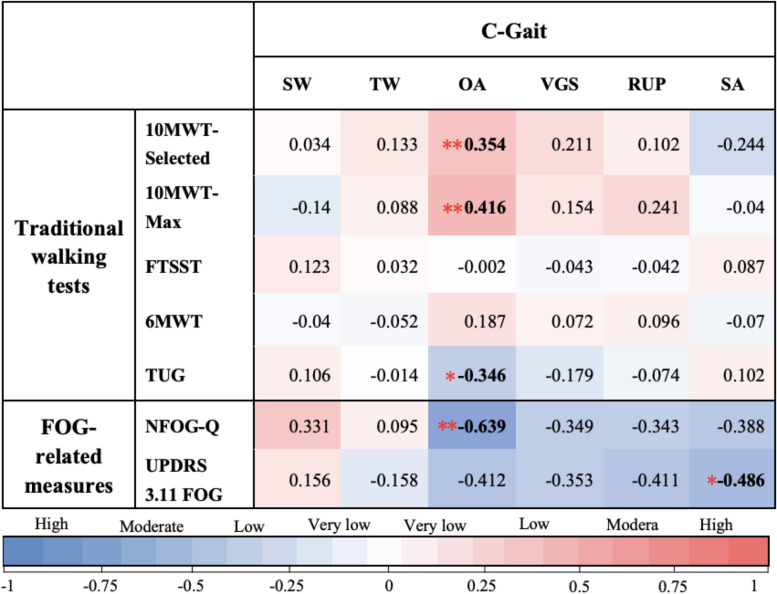
Spearman’s correlation coefficients between C-Gait assessment scores. In this study, correlation coefficients of 0–0.25, 0.25–0.50, 0.50–0.75, and 0.75–1.00 were considered to indicate very low, low, moderate, and high correlations, respectively. Slalom walking (SW), tandem walking (TW), obstacle avoidance (OA), visually guided stepping (VGS), reaction to unexpected perturbation (RUP), and speed of adaptation (SA) were on the *x*-axis, while traditional walking test measures were on the *y*-axis, i.e., The New Freezing of Gait Questionnaire (NFOG-Q), Unified Parkinson’s Disease Rating Scale Part III, item 11, and Freezing of Gait (UPDRS 3.11 FOG). Color types and shades provide a visualization of the direction and strength of the correlations, respectively. The numbers shown in each square correspond to different colors, indicating the Spearman’s correlation coefficient (r_*s*_) from Spearman’s rank correlation analysis of each pair. * Correlation is statistically significant at the *P* < 0.05 level; ** Correlation is statistically significant at the *P* < 0.01 level.

In C-Gait assessment items and traditional walking tests, freezers generally scored lower than non-freezers, with significant differences in obstacle avoidance, visually guided stepping, and speed of adaptation (Table 2; *P* < 0.05). Using Spearman’s rank correlation coefficient, by analyzing the correlation between C-Gait assessment items and walking-related clinical tests, we tested the validity of the C-Gait assessment to explore the feasibility of the C-Gait assessment. In this study, correlation coefficients between 0–0.25, 0.25–0.50, 0.50–0.75 and 0.75–1.00 were regarded as very low, low, moderate, and high correlations, respectively. Of the 30 possible correlations of C-Gait assessment score with walking function-related indexes, only three (10.0%) were significant. Although TUG (*r*_*s*_ = −0.346, *P* = 0.011), 10-MWT-Selected (*r*_*s*_ = 0.354, *P* = 0.009), and 10-MWT-Max (*r*_*s*_ = 0.416, *P* = 0.002) were significantly associated with obstacle avoidance, C-Gait assessment items had no to low correlation with traditional walking tests overall (Figure 1; *P* < 0.05).

**TABLE 2:** Differences between freezers and non-freezers in C-Gait assessment scores and clinical tests measures.

Variable	Total subjects (*N* = 53)	Freezers (*N* = 19)	Non-freezers (*N* = 34)	*P*
**The C-Gait assessment (score)**				
Slalom walking	7.55 ± 0.57	7.37 ± 0.76	7.65 ± 0.41	0.296*
Tandem walking	7.55 ± 0.59	7.39 ± 0.82	7.64 ± 0.40	0.521*
Obstacle avoidance	6.80 ± 0.90	6.31 ± 1.06	7.07 ± 0.67	0.009*
Visually guided stepping	7.05 ± 0.77	6.75 ± 0.85	7.22 ± 0.68	0.038*
Reaction to unexpected perturbation	6.18 ± 1.09	5.83 ± 1.36	6.37 ± 0.86	0.150*
Speed of adaptation	6.83 ± 1.09	6.55 ± 0.75	6.99 ± 0.29	0.027*
**Traditional walking tests**				
10-MWT- Selected (m/s)	1.13 ± 0.19	1.07 ± 0.20	1.16 ± 0.18	0.270*
10-MWT-Max (m/s)	1.49 ± 0.23	1.41 ± 0.25	1.54 ± 0.21	0.072*
FTSST (s)	10.93 ± 3.14	11.33 ± 4.20	10.70 ± 2.41	0.889*
6MWT (m)	459.92 ± 86.79	448.16 ± 95.45	466.50 ± 82.32	0.704*
TUG (s)	9.98 ± 3.36	11.12 ± 5.12	9.34 ± 1.51	0.262*

Data are mean ± standard deviation. The 10-Meter Walking Test at the self-selected speed (10-MWT-Selected), 10-Meter Walking Test at the maximum speed (10-MWT-Max), Five-Times-Sit-to-Stand Test (FTSST), 6-Minute Walk Test (6-MWT), and Timed Up-and-Go test (TUG) were performed. *P < 0.05.

### Correlation of C-Gait Assessment With Freezing of Gait-Related Indexes in Freezers

Of the 12 possible correlations, only two C-Gait assessment items were significantly correlated with FOG-related measures, including obstacle avoidance (*r*_*s*_ = −0.639, *P* = 0.003) and speed of adaptation (*r*_*s*_ = −0.486, *P* = 0.035) (Figure 1).

### Freezing of Gait Detection by Various Models

In DM1 involving traditional walking tests, a discriminating variable predicted to contribute significantly (10-MWT-Max) was extracted. Then, a canonical discriminant function was established:

logit⁢(p)⁢1=3.715-2.963* 10⁢MWT-Max

In DM2 involving C-Gait assessment (Wilks’ lambda = 0.757, *P* = 0.001; canonical correlation = 0.493), obstacle avoidance was predicted to contribute significantly. Then, a canonical discriminant function was established:

logit⁢(p)⁢2= 8.033-1.255*obstacle

**FIGURE 2:**
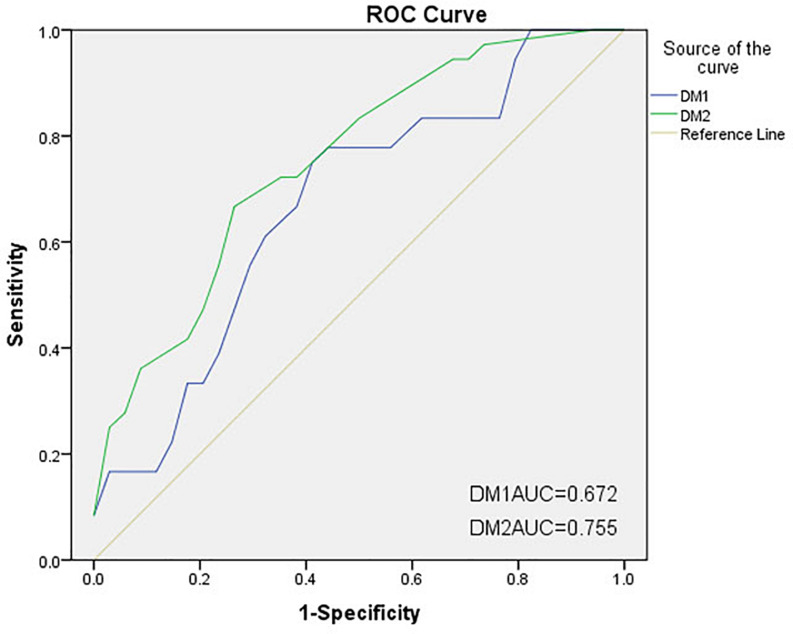
Receiver operating characteristic (ROC) curves of two discriminant models for freezing of gait (FOG). The areas under the ROC curves (AUCs) for DM1 and DM2 were 0.672 and 0.755, respectively. DM1, discriminant model 1 (traditional walking tests); DM2, discriminant model 2 (C-Gait assessment).

Two ROC curves were constructed based on the above formulas (Figure 2). The AUC for DM2 was 0.755, which was higher than that of DM1 (AUC = 0.672, *P* = 0.3312). The positive and negative predictive values for DM1 were 0.429 and 0.667, respectively, versus 0.700 and 0.738 for DM2, respectively.

### Between-Group Differences in Success Rates of Obstacle Avoidance

Three subjects (one freezer and two non-freezers) had missing raw data for obstacle avoidance, thus were not analyzed. In the remaining subjects, freezers (*N* = 18) and non-freezers (*N* = 32) showed a significant difference in the success rate of obstacle avoidance at the high difficulty level (level 4) (71% ± 20% vs. 83% ± 13%; *P* = 0.016) but not at the low difficulty level (level 2) (89% ± 15% vs. 93% ± 16%; *P* = 0.137).

## Discussion

This study demonstrated that C-gait assessment could provide additional value to the traditional walking tests for PD. Meanwhile, along the lines of gait adaptability, assessment may be able to discriminate between freezers and non-freezers.

Walking adaptability, the ability to modify walking to meet task goals and environmental demands (Grillner and Wallen, 1985), is required when the complexity of the walking task exceeds what can be achieved by the basic stepping. We referred to the framework of walking adaptability proposed by Balasubramanian et al. (2014) to provide a comprehensive picture of walking adaptability domains contained by C-Gait assessment.

In total, six of the seven C-Gait assessment items had walking adaptability demands, involving five domains of walking adaptability, and two items required the involvement of both domains (Supplementary Table 2). Therefore, we consider C-Gait assessment to be specific to walking adaptability and to reflect conditions in busy urban streets. Although it is not comprehensive enough and very suitable for PD patients who only needed to achieve walking on well-lit and flat roads, C-Gait assessment still has the potential to evaluate PD cases with demands to walk on complex environments. Therapists could expand walking adaptability domains simply by using external objects while performing basic C-Gait assessment.

C-Gait assessment could be applied to evaluate walking adaptability in the early to middle stages of PD as a complement to clinical tests. It could provide comprehensive quantitative data about the walking function in PD cases and help design appropriate rehabilitation therapy plans. In this study, no or low correlations between C-Gait assessment and traditional walking tests were found, corroborating previous studies (Geerse et al., 2018; Timmermans et al., 2019), and significant correlations mostly involved obstacle avoidance. This may be due to the fact that in current clinical treatment of PD, common walking function-related assessments usually focus on basic stepping and balance during steady-state walking but rarely involve the nine domains of walking adaptability (Supplementary Table 2). While they are valuable, it is difficult to provide a comprehensive picture of functional walking ability in PD cases and to measure the challenge to community living as well as the risk of adverse events such as falls. More importantly, because traditional walking tests and C-Gait assessments focus on different fields, no to low correlations could instead demonstrate that clinical tests and C-Gait assessment are complementary evaluation tools, indirectly suggesting that the latter is perfectly valid for walking adaptability. Interestingly, significant correlations mostly involved obstacle avoidance. This means that the obstacle avoidance task may have the potential to evaluate both steady-state walking function and walking adaptability. Unfortunately, no widely accepted “gold standard” for walking adaptability is currently available, and C-Gait assessment could not be directly examined for validity.

In this study, there was a correlation between FOG and poor performance on the two C-Gait assessment items including obstacle avoidance and speed of adaptation. In addition, obstacle avoidance was more valuable at the high difficulty level compared with the low difficulty level. Furthermore, obstacle avoidance was more sensitive than speed of adaptation in discriminating freezers from non-freezers.

Previous studies have found that PD patients perform poorly in response to sudden obstacles (Caetano et al., 2017), with the response speed decreasing with increasing task complexity (Cooper et al., 1994). Meanwhile, the shorter the available response time, the lower the success rate (Lu et al., 2019). Consistently, based on obstacle avoidance, this study showed that freezers had poorer walking performance due to higher demands on available response time for a high difficulty-level task. From the perspective of postural control, PD patients usually experience greater and faster mediolateral sway during obstacle crossing than healthy controls (Galna et al., 2013), and this impaired voluntary center of gravity (COG) control is more pronounced in the sagittal plane in those with FOG (Vervoort et al., 2013). In addition, sufficient visual information about the obstacle is crucial for successful obstacle avoidance, which can be used for advance planning and real-time modification of the obstacle avoidance behavior (Coolen et al., 2020). While a long obstacle may be perceived by PD cases as an external visual cue prompting increased step velocity and length during the lead crossing step, it does not seriously affect natural gait (Alcock et al., 2018). However, this is predicted to help achieve appropriate movement planning, unlike the condition in this study, where obstacles were not placed on the trajectory at the start of walking. Freezers were therefore unable to obtain sufficient feed-forward visual information (difficulty level negatively correlated with the amount of information provided) when confronted with an obstacle that suddenly appeared in front of them, nor could they adjust step length and speed with a proper strategy to avoid the obstacle through good control of the forward and backward shifts of COG. Meanwhile, the unpredictability of the timing of obstacle presentation, which requires additional attentional costs, combined with the failure feedback given after stepping on the obstacle, may exacerbate motor executive dysfunction and stress in freezers, thereby affecting walking performance. Of course, research on FOG has also evidenced that when freezers walk on a treadmill, sudden appearance of obstacles could provoke apparent FOG episodes (Snijders et al., 2010). The impaired stepping elicited by FOG could be a significant factor in score reduction in freezers.

For speed of adaptation, studies have demonstrated that when the energy demands of a motor task increase, patients with bradykinesia are more likely to move slowly (Mazzoni et al., 2007). Moreover, freezers showed poorer gait adjustment and longer adaptation times to sudden changes in belt speed compared with non-freezers, with aggravation under time pressure (Mohammadi et al., 2015). In this study, confronted with the abruptly changing and continuously accelerating walking area, freezers were required to not only constantly restrain anticipatory postural adjustments at the previous speed but also rapidly generate new gait patterns for the current speed. Therefore, under ongoing gait adjustments with speed, the computational load on pontomedullary reticular formation networks and the pedunculopontine nucleus could be further increased (Nonnekes et al., 2014), resulting in walking impairment in freezers. Consequently, freezers could not slow down significantly because of the belt and were forced to participate in subsequent acceleration tasks, resulting in decreased walking performance.

Although turning is also a condition eliciting FOG (Kader et al., 2017), freezers did not have a significant gait deficit in the slalom walking item in this study. This may be due to the fact that FOG is often provoked during the entire section of a full turn (360 degrees) at high speed (Spildooren et al., 2013). Meanwhile, the trajectory of subjects was set as a sinusoidal wave, to liken a combination of turning subtasks requiring continuous changes of direction but a small angle of turn each time. Even though this required to constantly execute turning movements, small rotations are not strict with demands in center of mass shifts and bilateral coordination, so freezers could maintain a steady walking speed not enough to reach the threshold for FOG episodes.

As shown above, visually guided stepping and tandem walking had no sensitivity in FOG detection likely because freezers considered the stepping target a visual cue, thus correcting their inefficiencies in visual exploration while walking (Hunt et al., 2018). The width of the walking area in the tandem walking task may not be narrower than the usual stride width of freezers, so they do not need to make complex gait adjustments.

Notably, in this study, the reaction to the unexpected perturbation item, similar to the obstacle avoidance item, showed no significance in FOG detection. That may be explained by the floor effect. This item required subjects to correctly respond to three changes in projected targets (the target randomly became an obstacle, the target randomly shifted longitudinally or laterally, and an obstacle suddenly appeared in the front walking surface). Meanwhile, PD patients have reduced adaptability (Caetano et al., 2017). Therefore, these conflict situations were too challenging for both groups of subjects, exceeding their limits of gait adjustment.

In the C-Gait assessment discrimination model, obstacle avoidance and speed of adaptation were sensitive to FOG detection (i.e., the two variables that contributed most to the model, although no correction has been made for the univariate multiple testing), indicating that C-Gait assessment has certain application value in identifying FOG. Unfortunately, the inability to implement cognitive dual-task item limits our further understanding of C-gait evaluation and recognition of FOG (Matar et al., 2013).

This study had some limitations. First, small total sample size and uneven sample sizes between groups. The FOG ratio is 35.8% in our study, which is a little lower than the FOG prevalence of 38.2% mainly because the patients’ enrollment criteria were different (Lloret et al., 2014). The higher UPDRS-III always exists in People with PD (PWP) with FOG compared to non-FOG when the patients were on the same H-Y stage (Choi et al., 2019). The imbalance possibly affected the discriminatory effect and caused bias. Secondly, whether FOG was triggered was not recorded, as well as the times and durations of FOG episodes. FOG provocation tasks were not applied in this study. All these need to be explored in a further study. Thirdly, C-Gait assessment could only simulate obstacles of different depths (e.g., puddles) but not those requiring the subjects to step over spatial obstacles; this problem may be solved by the recently emerged Microsoft HoloLens mixed-reality headset (Coolen et al., 2020). Fourthly, motor switching is extremely sensitive to dopamine (Cools et al., 2003), and PD patients in the “on” state were included in this study. Consequently, drug effects at baseline may narrow the differences between freezers and non-freezers. Finally, decreased walking adaptability is strongly associated with falls, which is common in early PD (Caetano et al., 2017), and the relationship between C-Gait assessment and the risk of falls should be assessed in future studies.

## Conclusion

C-Gait assessment could provide additional walking function information to the traditional gait evaluation in early- to middle-stage PD. Specifically, the gait adaptability assessment, as measured by C-Gait, may be able to help identify freezers in a PD population.

## Data Availability Statement

The raw data supporting the conclusions of this article will be made available by the authors, without undue reservation.

## Ethics Statement

The studies involving human participants were reviewed and approved by the Beijing Rehabilitation Hospital of Capital Medical University (No. 2018bkky022). The patients/participants provided their written informed consent to participate in this study.

## Author Contributions

BF and JX contributed to the study conception, design, and guidance. BF, H-JY, and Z-YC contributed to the definition of intellectual content. LQ and Q-XZ contributed to the literature search. H-JY, LQ, Q-XZ, and CL contributed to the clinical research. H-JY, LQ, Q-XZ, CL, J-PF, and YS contributed to the data collection. Z-YC, PW, Y-HL, R-DW, and Y-JL contributed to the data acquisition. Z-YC, LQ, Q-XZ, CL, J-PF, YS, X-YY, and A-XL contributed to the data analysis. BF, X-YY, PW, Y-HL, R-DW, and Y-JL contributed to the statistical analysis. BF, Z-YC, and H-JY contributed to the manuscript preparation. BF, JX, R-DW, Y-JL, J-PF, and YS contributed to the manuscript editing. BF, A-XL, and JX contributed to the manuscript review. All authors approved the final version of the manuscript.

## Conflict of Interest

The authors declare that the research was conducted in the absence of any commercial or financial relationships that could be construed as a potential conflict of interest.
