# Supplementary material for: C-Gait for Detecting Freezing of Gait in the Early to Middle Stages of Parkinson’s Disease: A Model Prediction Study
Source: Front Hum Neurosci. 2021 Mar 22;15:621977. doi: 10.3389/fnhum.2021.621977 (PMC8019899; doi:10.3389/fnhum.2021.621977)
Supplement: Supplementary file 1 [file Data_Sheet_1.PDF]

Supplementary Materials:

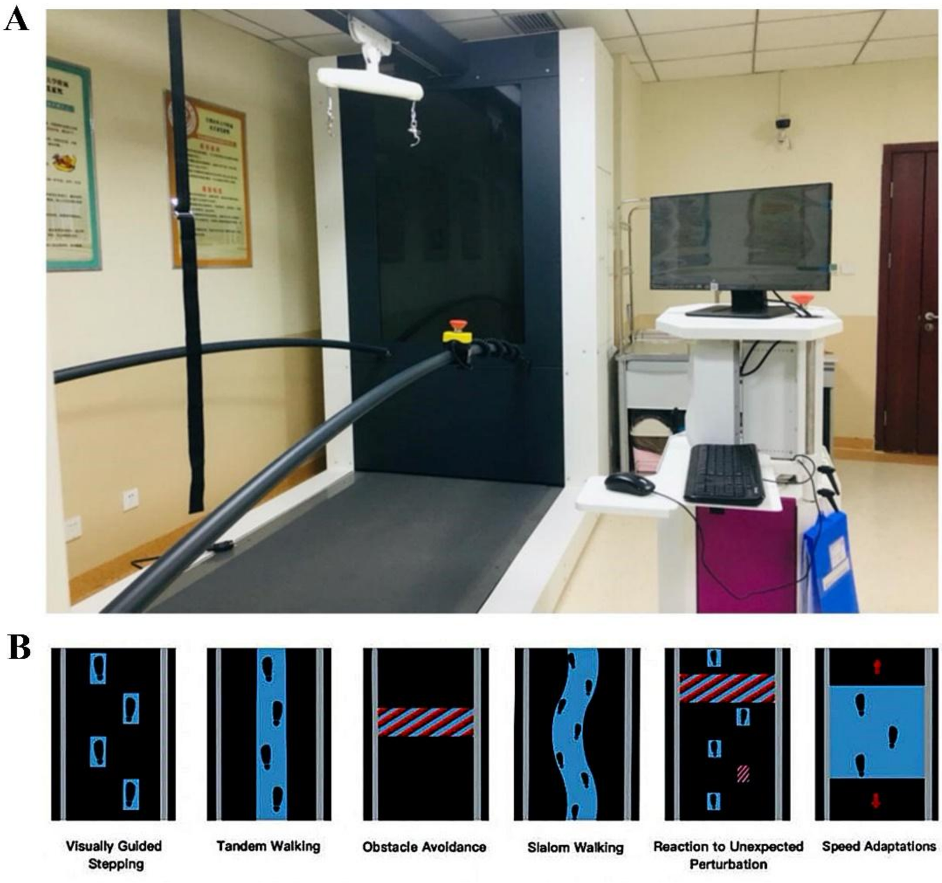

Supplementary Figure 1 : C-Mill and its assessment protocol. (A) The C-Mill of the neurorehabilitation center of Beijing Rehabilitation Hospital. (B) The six C-Gait assessment items included in this study: Visually guided stepping, the subjects must step on the auto projected target that matched the current gait pattern; Tandem walking, the subjects had to walk in the projection area on the belt; Obstacle avoidance, the subjects must avoid obstacles projected on the belt surface in front of them by crossing a full obstacle; Slalom walking, the subjects have to walk within the projected area on the belt (the projected slalom resembles a sine wave with the x-axis parallel to the vertical axis of the treadmill); Reaction to unexpected perturbation, the subjects are required to react to changing projected objects; Speed of adaptation, the subjects must walk within the projected square walking area, accelerated and decelerated up and down the treadmill.

Supplementary Table 1 Details of the 7 C-Gait assessment items

Items	Explanation
Visually guided stepping	Subjects must place their feet on the projected stepping target. Once a stable gait detected, the program automatically projects stepping targets that match the subject's current gait pattern.
Tandem walking	Subjects have to walk within the projected area on the belt, which is a long bar parallel to the longitudinal axis of the treadmill. The higher the difficulty level, the narrower the projected bar.
Obstacle avoidance	Subjects must avoid obstacles projected on the belt surface in front of them by crossing a full obstacle. The sizes of obstacles and the available response times vary depending on the difficulty level.
Slalom walking	Subjects have to walk within the projected area on the belt. The projected slalom resembles a sine wave with the x-axis parallel to the vertical axis of the treadmill. The higher the difficulty level, the higher the frequency of the sine wave.
Reaction to unexpected perturbation	Subjects must correctly respond to different projected objects that must be either avoided or hit. During this task, subjects are required to react to changing projected objects (the target randomly turned into an obstacle, the target randomly shifted longitudinally or laterally, and an obstacle suddenly appeared in the front walking area). The percentage of changing stepping objects varies depending on the difficulty level.
Speed of adaptation	Subjects must walk within the projected square walking area. This area accelerates and decelerates up and down the treadmill. The higher the difficulty level, the more the walking area accelerates. The deceleration is always equal.
Cognitive dual task	Subjects must walk following the rhythm of the auditory cue in the task. As soon as a steady gait pattern is detected, the auditory cue will start automatically.

C-Gait in this study consisted of 6 items. Each item was divided into 5 levels of difficulty (levels 1, 2, 3, 4, and 5). Among them, the highest level was 4, and the difficulty level could reach 5 in gait adaptability training. A C-Gait test consisted of two parts, evaluating the 6 items at the difficulty levels of 2 and 4, respectively. The C-Gait comprehensive score is based on difficulty level and performance: C-Gait score for each task=level*2*performance percentage/100; C-Gait baseline evaluation score=highest level (4)*2*percentage of performance/100.

Supplementary Table 2: Domains of Walking Adaptability Contained in the C-Gait Assessment Items

	Domains of Walking Adaptability Framework								
	ON	TM	CT	TR	AM	PT	MT	PL	TF
Visually guided stepping									
Tandem walking						✓			
Obstacle avoidance	✓								
Slalom walking						✓			✓
Reaction to unexpected perturbation	✓								✓
Speed of adaptation		✓							
Cognitive dual task			✓						

ON, obstacle negotiation; TM, temporal demands; CT, cognitive dual-tasking; TR, terrain demands; AM, ambient demands; PT, postural transitions demands; MT, motor dual-tasking; PL, physical load; TF, maneuvering in traffic.

Appendix 1 Increase in difficulty for each task

1. Goal-oriented walking: randomly increase step length and step width

2. Tandem walking: reduce the width of the walking area

3. Avoid obstacles: increase the size of the obstacle and reduce the available reaction time (ART)

4. Curve walking: increase the curvature of the curve

5. Obstacle disturbance response: increase the size of the obstacle and reduce the available reaction time (ART)

6. Walking speed adjustment: increase the acceleration of the walking area

The higher the level of difficulty in the assessment, the more difficult it is to complete the task, and the more it can reflect gait adaptability in PD patients.
